# The low FODMAP diet in adolescents functional abdominal in a non-guided setting: a prospective multicenter cohort study

**DOI:** 10.1007/s00431-025-05999-9

**Published:** 2025-02-11

**Authors:** Robyn Rexwinkel, Nicolaas Koen Vermeijden, Judith Zeevenhooven, Johannes Kelder, Michael Groeneweg, Thalia Hummel, Joery Goede, Herbert van Wering, Janneke Stapelbroek, Marc Benninga, Arine Vlieger

**Affiliations:** 1 Emma’s Children Hospital, Amsterdam UMC, Location AMC, University of Amsterdam, Pediatric Gastroenterology, Hepatology and Nutrition, Room C2-312, PO Box 22700, 1100 DD Amsterdam, The Netherlands; 2 Amsterdam Reproduction & Development Research Institute, Amsterdam University Medical Centers, Location Academic Medical Center/Emma Children’s Hospital, Amsterdam, The Netherlands; 3 Gastroenterology and Hepatology, Amsterdam Gastroenterology Endocrinology Metabolism Research Institute,, Amsterdam UMC, University of Amsterdam, Amsterdam, The Netherlands; 4 Department of Pediatrics, St Antonius Hospital, Nieuwegein, The Netherlands; 5 Department of Cardiology, St Antonius Hospital, Nieuwegein, The Netherlands; 6 Department of Pediatrics, Maasstad Hospital, Rotterdam, The Netherlands; 7 Department of Pediatrics, Medisch Spectrum Twente, Enschede, The Netherlands; 8 Department of Pediatrics, Spaarne Gasthuis, Haarlem, The Netherlands; 9 Department of Pediatrics, Amphia Hospital, Breda, The Netherlands; 10 Department of Pediatrics, Catharina Hospital, Eindhoven, The Netherlands

**Keywords:** FODMAP, Functional abdominal pain disorders, Children

## Abstract

The purpose of this study is to evaluate the efficacy of a diet low in fermentable oligosaccharides, disaccharides, monosaccharides, and polyols (FODMAPs) in adolescents with irritable bowel syndrome (IBS) and functional abdominal pain-not otherwise specified (FAP-NOS) in a non-guided setting, resembling clinical practice. This prospective multicenter cohort study conducted in 13 centers included patients aged 12–18 years diagnosed with IBS or FAP-NOS. Patients received educational material on FODMAPs, including extensive lists of high and low FODMAP foods and additional online information. They were instructed to replace high FODMAP foods with low FODMAP alternatives for the duration of 4 weeks. No dietician was consulted. The primary end point was the proportion of patients with treatment success (≥ 30% reduction of abdominal pain intensity) at 4 weeks. The key secondary outcome was adequate relief of IBS/FAP-NOS symptoms. Of the 325 included patients, 81 patients (24.9%) achieved treatment success (≥ 30% reduction of abdominal pain intensity) after 4 weeks, with higher rates in patients with IBS (29.3%) than FAP-NOS (16.8%, OR 2.16 (1.04–4.48)). Adequate relief was reported in 51 patients (15.7%). There was a significant decrease in abdominal pain intensity (2.2 (1.1) vs. 2.5 (1.0),* P* < 0.001), daily bloating (2.4 (2.1) vs. 2.8 (2.3), *P* < 0.001), and flatulence (2.4 (2.1) vs. 2.8 (2.3), *P* = 0.001). Adverse events were mild and infrequent.

*Conclusion*: The low FODMAP diet in a non-guided setting, mimicking clinical practice, yielded treatment success in almost 30% adolescents with IBS and 17% in FAP-NOS, suggesting it may not be the first treatment option for these patients.

*Trial registration*: EUCTR2015-003293–32-NL.

**Table:** 

**What is Known:** • *Irritable bowel syndrome (IBS) and functional abdominal pain-not otherwise specified (FAP-NOS) are common disorders in children which negatively impact quality of life.* • *While a diet low in fermentable oligosaccharides, disaccharides, monosaccharides, and polyols (FODMAPs) has demonstrated effectiveness in adult IBS, its efficacy in pediatric IBS and FAP-NOS remains uncertain.* • *Clinical application of the low FODMAP diet often occurs without dietician consultation, contrary to controlled trial settings.* **What is New:** *• The low FODMAP diet, without dietician guidance, resulted in treatment success in almost 30% of adolescents with IBS and only 17% with FAP-NOS.* *• With only 15.7% of participants achieving adequate relief of IBS/FAP-NOS symptoms, the non-guided low FODMAP diet may not be the first treatment option for pediatric IBS and FAP-NOS.*

**Supplementary Information:**

The online version contains supplementary material available at 10.1007/s00431-025-05999-9.

## Introduction

Irritable bowel syndrome (IBS) and functional abdominal pain-not otherwise specified (FAP-NOS) are disorders of the gut brain interaction, characterized by chronic or recurrent abdominal pain, without evidence of an underlying organic disorder. IBS is characterized by altered bowel habits and pain related to defecation, as opposed to FAP-NOS, in which the pain is not associated with defecation [1, 2]. IBS and FAP-NOS are highly common in childhood with a worldwide pooled prevalence of approximately 10% [3]. The current pathophysiology remains uncertain. Contributing factors include alterations in neurohumoral mechanisms, psychosocial disturbances, genetic factors, abnormal gastrointestinal motility, and visceral hypersensitivity [4–8].

These prevalent disorders are of particular concern, as they are associated with a reduced quality of life (QoL), an increased risk for anxiety and depression disorders and an increase in school absenteeism [9–12]. IBS and FAP-NOS, therefore, impose a substantial emotional and financial burden [13].

Currently, there is insufficient evidence to recommend any drug for the treatment of pediatric IBS or FAP-NOS [14]. Consequently, patients and health care professionals often discuss non-pharmacological approaches, such as diets or psychological therapies [15]. In recent years, a diet low in fermentable oligosaccharides, disaccharides, monosaccharides, and polyols (FODMAPs) has gained popularity [16, 17]. The rationale behind this diet is that unabsorbed and non-digested FODMAPs increase small intestinal water content and undergo fermentation in the colon, leading to gas production and intestinal distension. This process occurs in both IBS patients and healthy controls. However, due to visceral hypersensitivity in a subset of IBS patients, this distension can result in abdominal pain in patients, but not in healthy subjects [16, 18].

While there is a growing body of evidence supporting the efficacy of a low FODMAP diet as treatment option for adult patients with IBS, the evidence of its effectiveness in pediatric IBS and FAP-NOS is limited and inconsistent [16, 19]. A systematic review on the low FODMAP diet in pediatric IBS and FAP-NOS concluded that there is insufficient evidence for or against the efficacy of the low FODMAP diet. This review included five studies with a total of 153 patients (5–18 years old) with intervention periods ranging from 2 days to 2 months. Unfortunately, none of the included studies used the recommended outcome measures as advised by the Rome guideline for the design of clinical trials in children [19–21]. Moreover, these study designs included prepared meals (*n* = 4) or strict guidance of a dietician (*n* = 1). This approach differs from clinical practice, where patients are often advised by their treating physician to seek more detailed information on the low FODMAP diet online.

We aimed to assess the effect of the low FODMAP diet in a non-guided setting, thereby mimicking clinical practice, in a large patient group, using the recommended Rome outcome measures [20].

## Methods

### Study design and patient selection

This was a prospective multicenter cohort study conducted in 13 centers (1 academic and 12 non-academic) in The Netherlands. From November 2018 through September 2022, patients aged 12 to 18 years diagnosed with IBS or FAP-NOS according to the ROME IV criteria were invited to participate. During a period of 1 week prior to inclusion, eligible patients were required to have an average daily pain score of ≥ 2 on a scale from 0 to 5, where 0 represents no pain and 5 the worst imaginable pain [1, 20]. Screening involved history-taking, physical examination, and, based on their treating physician’s discretion, routine laboratory testing to rule out underlying organic diseases. Exclusion criteria included concurrent treatment by another healthcare professional for abdominal pain, the presence of organic gastrointestinal diseases, use of drugs affecting gastrointestinal motility, and insufficient knowledge of the Dutch language. Written informed consent was obtained from all patients before initiating any trial-specific procedure.

The trial was conducted in accordance with the principles of Good Clinical Practice and the Declaration of Helsinki and registered with the International Clinical Trials Registry Platform (NL7508).

### Intervention

All patients received explanation of the nature of their abdominal pain, as well as a clarification on how FODMAPs can induce gastrointestinal symptoms by their treating physician. All patients were given written patient information forms. These materials, developed by dietitians from a Dutch hospital, provide comprehensive guidance on the FODMAP diet. They include explanations of FODMAPs, their role in abdominal symptoms, a detailed list of high-FODMAP foods with low-FODMAP alternatives, meal planning advice, FAQs, and references to reputable resources such as patient organization websites, the Dutch Food Centre, and the Monash University Low FODMAP Diet app. Supplementary appendix 1 and 2 contain these forms in Dutch. Patients were instructed to eliminate high FODMAP foods from their diet and recommended to substitute them with low FODMAP alternatives for a duration of 4 weeks. This timeframe aligns with clinical practice recommendations, allowing sufficient time for the majority of patients to experience symptom improvement [22]. During the study period, no dietician was consulted.

### Efficacy endpoints

The primary endpoint was the proportion of patients achieving treatment success, defined as a ≥ 30% reduction of their abdominal pain intensity after 4 weeks of dietary intervention as compared to baseline [20]. This was assessed by a standardized pain diary card sent to participants, on which they recorded daily intensity and frequency of abdominal pain during a period of 7 consecutive days. Pain intensity was scored using the Wong Baker Faces scale [23]. This is a validated pain scale to measure pain intensity, with 0 indicating no pain and 5 the worst imaginable pain. These abdominal pain diaries were completed at baseline (T0) and at the end of the intervention period (T1 = 4 weeks).

The key secondary endpoint was the proportion of patients with adequate relief of IBS/FAP-NOS symptoms 4 weeks after start of the dietary intervention. Patients and/or parents were asked a single question (Did you/your child have adequate relief of IBS/FAP-NOS symptoms (abdominal discomfort/pain, bowel habits, and other symptoms like nausea and bloating) over the past week?). This was scored on a dichotomous scale (Yes/No). Other secondary endpoints were changes in mean pain frequency (as scored in hours of abdominal pain per day), bloating, flatulence, headache, nausea and loss of appetite (all scored on a 0–10 scale), and the number of days with school absence or use of pain medication. These endpoints were also recorded in the standardized pain diary card sent to participants.

Baseline characteristics were collected for secondary multiple logistic regression analysis to identify baseline predictors for treatment success and adequate relief at T1. These included sex, age, ethnicity, diagnosis, duration of symptoms, family history of functional abdominal pain disorders (FAPDs), and expectations of treatment success rated by patients and parents (scored on a 10-point Likert scale, with 0 indicating no expected improvement and 10 the best improvement). Secondary logistic regression analysis also was performed to identify specific symptom improvement that are associated with treatment success or adequate relief at T1. Supplementary Table 1 lists the endpoints used.

### Statistical analysis

The sample size was exploratory. All patients eligible and willing to participate were included.

All efficacy and safety analyses were performed on an intention-to-treat population, which included all patients of whom any data was collected. Missing data from standardized diaries were imputed with the use of multiple imputation. Twenty cycles of imputation were performed, with the assumption that unobserved measurements were missing at random. Two sensitivity analyses were conducted: a “worst-case” analysis in which missing data were replaced with the worst value (e.g., highest abdominal pain score) and a “best-case” analysis in which missing data were replaced with the best value (e.g., no abdominal pain).

Descriptive statistics were used to summarize variables. Continuous variables with normal distribution were expressed as mean with standard deviation. Variables with non-parametric distribution were expressed as median with interquartile range (IQR). Mean change in endpoints was assessed with paired samples *T*-test with significance set at *α* = 0.005 after Bonferroni correction. Baseline characteristics, that in a univariate analysis were associated with treatment success (*P* < 0.20) or that were chosen based on pathophysiological insights, were entered in a multiple logistic regression model. This secondary multiple logistic regression analysis identified baseline predictors for treatment success and adequate relief at T1. Another secondary multiple logistic regression analysis was performed to identify specific symptom improvement associated with treatment success and adequate relief at T1. Safety assessments included the number of adverse and serious adverse events. Statistical analyses were performed using SPSS version 28.0 (IBM Corporation, Armonk, NY).

### Ethics and dissemination

The medical ethics committee of the Amsterdam UMC (MEC AMC 018) approved the study. Moreover, the study was approved by the local ethics committees of all recruiting sites.

## Results

### Patients

**Fig. 1:**
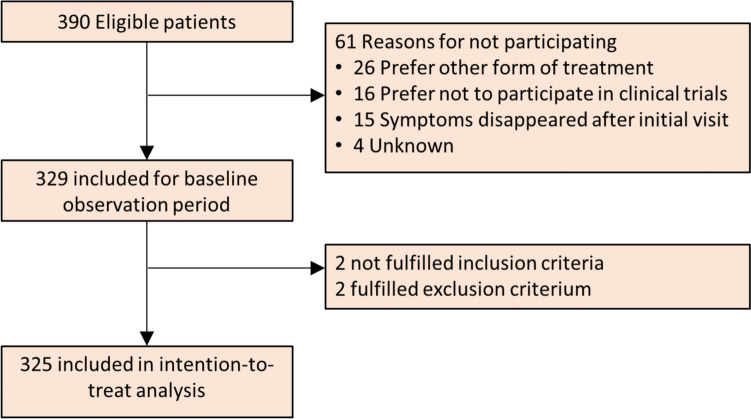
Flowchart of participants

From November 2018 to September 2022, a total of 390 patients were screened. Of these, 329 patients were included, after which four were excluded from analyses for fulfilling an exclusion criterion. A total of 325 patients completed the 4-week study and were included in the intention-to-treat population (Fig. 1).

At baseline, the mean age of the patients was 15.2 years (± 1.7), 229 (70%) were girls, and 212 (65%) of the patients had IBS compared to 112 (35%) diagnosed with FAP-NOS. The median duration of symptoms was 3 years (IQR 0.8–8.0). Mean digital diary compliance was 92.9% (interquartile range (IQR) 71.4–100.0%). Table 1 shows all demographic and baseline characteristics.

**Table 1:** Demographics and clinical characteristics

Characteristic	(*n* = 325)
Age, mean (SD), years	15.2 (1.7)
Female, frequency (%)	229 (70%)
Male	96 (30%)
Including center	
Secondary	163 (50%)
Tertiary	162 (50%)
Ethnicity, frequency (%)	
White	263 (81%)
Middle Eastern/North African	25 (8%)
Afro-Caribbean	17 (5%)
Other	20 (6%)
Diagnosis, frequency (%)	
FAP-NOS	113 (35%)
IBS	212 (65%)
Subtypes	
IBS-C	93 (29%)
IBS-D	56 (17%)
IBS-M	39 (12%)
IBS-U	22 (7%)
Duration of symptoms, median (IQR), years	3.0 (0.8–8.0)
First line family member with FAPD (%)	116 (36%)
Abdominal pain scores, mean (SD) þ	
Daily intensity score, mean (SD)	2.5 (1.0)
Daily frequency, hours, median (IQR)	5.3 (6.1)
Associated symptoms scores, mean (SD)	
Bloating	3.3 (2.4)
Flatulence	2.8 (2.3)
Nausea	2.3 (2.1)
Headache	2.5 (2.1)
Expectation of treatment, mean (SD) ‡	
Patient	5.7 (2.0)
Mother or parent 1	6.2 (1.7)
Father or parent 2	5.9 (1.9)

þ Abdominal pain intensity was scored on a scale from 0 to 5, with 0 indicating no pain and 5 the worst imaginable pain

‡ Expectation of treatment was scored on a scale from 0 to 10, with 0 indicating no expected improvement and 10 highest expected improvement

*FAP-NOS* functional abdominal pain-not otherwise specified, *IBS* irritable bowel syndrome, *IBS-C* irritable bowel syndrome with predominant constipation, *IBS-D* irritable bowel syndrome with predominant diarrhea, *IBS-M* irritable bowel syndrome with mixed bowel habits, *IBS-U* irritable bowel syndrome unclassified

### Efficacy

#### Primary endpoint

At week 4, out of the 325 included patients, 81 (24.9%) achieved treatment success, defined as a ≥ 30% reduction of abdominal pain intensity compared to baseline. Sensitivity analyses (i.e., “worst-case/best-case” analysis) to assess the effect of missing values from the abdominal pain diaries yielded different results. “Worst-case” analysis (e.g., missing values indicated highest abdominal pain score) resulted in treatment success in 70 participants (21.5%), while in the “best-case” analysis (e.g., missing values indicated no abdominal pain), treatment success was achieved in 129 participants (39.7%).

Treatment success was higher in patients diagnosed with IBS compared to FAP-NOS. Sixty-two of 212 (29.3%) IBS patients achieved treatment success, while 19 of 113 (16.8%) FAP-NOS patients experienced treatment success at week 4 (IBS vs. FAP-NOS OR 2.16 (1.04–4.48)).

Secondary multiple logistic regression identified no other baseline characteristics to be a predictor for treatment success. Treatment success was not modified by sex, age at inclusion, positive family history, IBS subtype, and duration or severity of abdominal pain and non-abdominal symptoms (Supplementary Table 2). Improvement of bloating, flatulence, defecation frequency, headaches, nausea, and loss of appetite were not associated with treatment success, while improvement of abdominal pain frequency in hours was associated with treatment success at T1 (Supplementary Table 3).

### Secondary endpoints 

#### Adequate relief

After 4 weeks, 51 patients (15.7%) reported adequate relief of their IBS/FAP-NOS symptoms. Adequate relief rates were similar between patients with IBS (*n* = 37 (17.5%)) and FAP-NOS (*n* = 14 (12.4%)) (OR 1.50 (0.77–2.90)). Secondary multiple logistic analysis revealed that the probability of achieving adequate relief was higher for girls and patients with lower abdominal pain intensity at baseline. Other baseline characteristics were not associated with adequate relief of IBS/FAP-NOS symptoms (Supplementary Table 4). Improvement of abdominal pain severity and duration, bloating, flatulence, headaches, nausea, and loss of appetite was not associated with treatment success, while a lower defecation frequency was associated with treatment success at T1 (data not shown).

### Other secondary endpoints

At week 4, there was a significant decrease in mean daily abdominal pain intensity compared to baseline (2.2 (1.1) vs. 2.5 (1.0), *P* < 0.001). Furthermore, patients recorded less daily bloating (2.4 (2.1) vs. 2.8 (2.3), *P* < 0.001), less flatulence (2.4 (2.1) vs. 2.8 (2.3), *P* = 0.001), and harder stool consistency (4.1 (0.2) vs. 4.0 (0.3), *P* < 0.001). These improvements were consistent regardless of IBS or FAP-NOS diagnosis. No differences were recorded with respect to number of days with school absence or use of painkillers, daily headache, nausea, loss of appetite, and defecation frequency (Table 2).

**Table 2:** Secondary endpoints at baseline and after 4 weeks of dietary intervention

	T1 (week 0)	T2 (week 4)	*P*-value
Daily intensity score, mean (SD) þ	2.5 (1.0)	2.2 (1.1)	< 0.001
Daily frequency, hours, mean (SD)	5.3 (6.1)	5.0 (5.9)	0.144
Bloating, severity, mean (SD) Ж	3.3 (2.4)	2.7 (2.2)	< 0.001
Flatulence, severity, mean (SD) Ж	2.8 (2.3)	2.4 (2.1)	0.001
Defecation frequency per day, mean (SD) ͱ	3.4 (1.6)	3.6 (1.7)	0.052
Defecation consistency, mean (SD)	4.0 (0.3)	4.1 (0.2)	< 0.001
Headache, severity, mean (SD) Ж	2.5 (2.1)	2.3 (2.0)	0.399
Nausea, severity, mean (SD) Ж	2.3 (2.1)	2.1 (2.0)	0.216
Loss of appetite, severity, mean (SD) Ж	2.0 (2.1)	1.9 (1.9)	0.504
Use of painkillers, days, mean (SD) ꟸ	1.0 (1.5)	1.3 (1.6)	0.071
School absence, days, mean (SD) ¤	1.0 (1.5)	1.2 (1.5)	0.464

﻿þ Abdominal pain intensity was scored on a scale from 0 to 5, with 0 indicating no pain and 5 the worst imaginable pain

Ж Bloating, flatulence, headache, nausea, and loss of appetite were scored on a scale from 0 to 10, with 0 indicating no nausea, headache, or loss of appetite and 10 the highest severity of these symptoms

ꟸ Use of painkillers was scored as the number of days of that week that the patient took a painkiller

ͱ Defecation consistency was scored using the Bristol stool scale, with 1 indicating very hard stool and 7 indicating very loose stool

¤ School absence was scored as the number of days of that week that the patient was absent from school

### Safety

Adverse events were mild and infrequent. No serious adverse events were recorded (Supplementary Table 5).

## Discussion

In this prospective, a multicenter cohort study involving children with IBS or FAP-NOS, a non-guided low FODMAP diet setting, thus mimicking clinical practice, resulted in 24.9% of patients achieving treatment success, defined as ≥ 30% reduction of abdominal pain intensity. Adequate relief was achieved in only 15.7% of the patients. IBS patients were more likely to achieve treatment success compared to FAP-NOS patients; however, adequate relief rates were similar between both diagnoses. Girls and patients with lower abdominal pain intensity at baseline were more likely to achieve adequate relief of IBS-FAP-NOS symptoms.

Comparison of our treatment success rate of 24.9% with previous studies on low FODMAP diets in pediatric IBS or FAP-NOS is hampered by the fact that these studies used different outcome measures [19, 20]. Data on mean abdominal pain intensity change from other studies is available, yet inconclusive. One study reported no significant change in abdominal pain intensity, whereas two studies reported a significant decrease, aligning with our observed decrease in pain intensity [24–26]. However, our observed reduction from 2.5 (± 1.0) to 2.2 (± 1.1), while statistically significant, may lack clinical significance due to its relatively small magnitude.

In contrast to our results, adult studies, incorporating the same definition of treatment success, revealed higher rates of treatment success (51–60%) and adequate relief (52–61%) [27–31]. These differences may be caused by the comprehensive dietary consultation provided in adult studies, mostly conducted by specially trained dietitians, alongside the well-observed high dietary adherence. In contrast, the present study provided only brief explanation of the low FODMAP diet with written instructions and treatment adherence was not monitored. It is well known that treatment compliance in adolescents tends to be noteworthy low [32].

In the present study, treatment success was significantly higher in patients diagnosed with IBS compared to FAP-NOS. Research suggests that the gut microbiota in IBS patients has an elevated capacity to utilize fermentable carbohydrates. Reliance of IBS-related bacteria on this energy substrate could explain why a diet low in fermentable carbohydrates, such as the low FODMAP diet, can result in symptom improvement [33]. Moreover, in another study in a pediatric IBS population, responders to the low FODMAP diet demonstrated a different microbiome profile characterized by greater saccharolytic potential [26]. The difference in low FODMAP efficacy between IBS and FAP-NOS patients may stem from different microbiome profiles. However, this hypothesis remains to be investigated.

Adequate relief of IBS/FAP-NOS symptoms was more frequently reported by patients presenting with lower abdominal pain intensity and by girls. This first observation could partially be explained by the natural course of the disease with patients with less severe complaints showing more often a spontaneous improvement of their symptoms. The higher incidence of postprandial gas problems and abdominal pain in females, mostly related to carbohydrate intake including FODMAPs, might explain the higher adequate relief rates in females [34]. Notably, pediatric literature on low FODMAP efficacy lacks comprehensive data on baseline predictors, whereas adult studies present mixed findings. Two studies did not identify any predictors for treatment success [27, 35]. A Swedish study, however, reported treatment success more often in females, while in a Chinese cohort, not gender, but more severe IBS symptoms at baseline were correlated with higher likelihood of treatment success [36, 37]. Given these contradictory findings, additional studies are warranted to identify baseline predictors of treatment success and adequate relief.

Our cohort observed a statistically significant, albeit likely clinically insignificant reduction in bloating and flatulence as well as harder stools with the low FODMAP diet. The reduction was less pronounced than in adult studies, where mean decreases ranged from 11.7 to 22.9 on a 0–100 scale [27, 31, 35, 36, 38–40]. These differences in effect size on bloating and flatulence may be attributed to differences in study design as discussed above. It is well known that unabsorbed and non-digested FODMAPs increase small intestine’s water content and undergo fermentation in the colon, leading to gas production and intestinal distension—manifestations commonly linked to bloating and flatulence [16, 18]. However, our analysis indicated that the reduction of bloating and flatulence was not the primary contributor to the overall alleviation of abdominal pain. This suggests that while FODMAPs indeed influence bloating and distention, their impact on abdominal pain might operate through other mechanisms.

Strengths of our study include the largest population to date in pediatric FODMAP trials and the effort to replicate a clinical setting by not providing prepared meals or extensive dietary guidance. Moreover, this is the first study to adopt the recommended treatment success definition of ≥ 30% reduction in abdominal pain intensity [20]. Additionally, we incorporated the advised 4-week FODMAP restriction, which balances symptom improvement with potential negative impacts on microbiota and nutritional adequacy [22].

An important limitation of our study is the absence of a control group, making it challenging to attribute the outcomes solely to the dietary intervention. Natural variability, regression to the mean, or the placebo effect may have influenced results. The lack of dietary supervision may have reduced treatment success, as a dietician could provide enhanced support, customization, and education. Additionally, external validity is constrained by the inclusion of only adolescents. Following Dutch clinical practice, we only verbally assessed adherence to the FODMAP diet. However, treatment adherence in adolescents tends to be noteworthy low, which may have contributed to lower treatment efficacy [32]. It also remains unclear whether patients searched for additional information online or whether variations in habitual diet may have affected treatment responses. Individuals who responded to the intervention might have had higher initial FODMAP intake, suggesting the need for more personalized approaches. Finally, no data were collected on potentially relevant adverse events, such as weight loss, restrictive eating habits, or nutritional status. However, the duration of the dietary aligned with recommendations to minimize potential negative effects on microbiota and nutritional adequacy [22]. Future research should focus on a non-guided low FODMAP diet compared to a control group receiving standard care or a diet without therapeutic effect, while measuring treatment adherence and using recommended outcome measures.

In conclusion, the low FODMAP in a non-guided setting resulted in treatment success in only 29.3% of adolescents with IBS and 16.8% with FAP-NOS. This suggest that the FODMAP diet might be less effective than other proven treatment options for IBS and FAP-NOS, such as hypnotherapy and cognitive behavioral therapy [41, 42]. Therefore, the low FODMAP diet may not be the first treatment option to recommend, especially not in adolescents with FAP-NOS. However, no definite recommendations can be made based on the results of this study.

## Supplementary Information

Below is the link to the electronic supplementary material.

Supplementary file1 (DOCX 37 KB)

Supplementary file2 (PDF 3.14 KB)

Supplementary file3 (PDF 212 KB)

## Data Availability

The datasets generated and analyzed during the current study are available upon request.

## Abbreviations

FODMAPs: Fermentable oligosaccharides, disaccharides, monosaccharides, and polyols
FAP-NOS: Functional abdominal pain-not otherwise specified
FAPDs: Functional abdominal pain disorders
IBS: Irritable bowel syndrome
QoL: Quality of life
