# Supplementary material for: The low FODMAP diet in adolescents functional abdominal in a non-guided setting: a prospective multicenter cohort study
Source: Eur J Pediatr. 2025 Feb 11;184(2):189. doi: 10.1007/s00431-025-05999-9 (PMC11814023; doi:10.1007/s00431-025-05999-9)
Supplement: Supplementary file 1 — Supplementary file1 (DOCX 37 KB) [file 431_2025_5999_MOESM1_ESM.docx]

**Supplementary table 1** Overview of endpoints

| Endpoint | Instrument | T0 | T1  4 weeks after start treatment |
| --- | --- | --- | --- |
| Abdominal pain | Abdominal pain diaries | **X** | **X** |
| Associated symptoms | Abdominal pain diaries | **X** | **X** |
| Defecation frequency and consistency | Abdominal pain diaries | **X** | **X** |
| School absence | Abdominal pain diaries | **X** | **X** |
| Use of pain medication | Abdominal pain diaries | **X** | **X** |
| Adequate relief | Binary question on adequate relief |  | **X** |

**Supplementary table 2** Logistic regression analysis to identify baseline factors predicting treatment success at T1.

| Predictor* | Simple logistic regression | | Multiple logistic regression | |
| --- | --- | --- | --- | --- |
|  | **OR (95% CI)** | **p-value** | **OR (95% CI)** | **p-value** |
| Sex (female vs male) | 1.21 (0.63 – 2.31) | .570 | 1.25 (0.62 – 2.52) | .536 |
| Age at inclusion (in years) | 1.03 (0.87 – 1.21) | .771 |  |  |
| Ethnicity  White (reference)  Middle Eastern/North African  Afro-Caribbean  Other | 1.07 (0.86 – 1.32)  0.92 (0.72 – 1.17)  1.00 (0.76 – 1.31) | .550  .483  .975 |  |  |
| First-line family member with FAPD | 1.23 (0.62 – 2.41) | .555 | 1.26 (0.38 – 2.30) | .463 |
| Diagnosis (IBS vs. FAP-NOS) | 2.16 (1.04 – 4.48) | .038 | 2.52 (1.15 – 5.56) | .022 |
| IBS subtype  IBS-U (ref)  IBS-C  IBS-D  IBS-M | 1.85 (0.53 – 6.51)  2.03 (0.50 – 8.30)  1.48 (0.35 – 6.29) | .337  .323  .597 |  |  |
| Duration of symptoms (in years) | 1.00 (0.92 – 1.08) | .943 |  |  |
| Including center (academic vs teaching center) | 1.53 (0.87 – 2.68) | .141 | 1.45 (0.78 – 2.68) | .238 |
| Mean abdominal pain severity at baseline | 0.98 (0.73 – 1.32) | .877 |  |  |
| Mean abdominal pain frequency at baseline | 0.97 (0.92 – 1.02) | .246 | 0.98 (0.93 – 1.04) | .981 |
| Mean bloating at baseline | 0.98 (0.87 – 1.11) | .778 |  |  |
| Mean flatulence at baseline | 1.01 (0.89 – 1.15) | .897 |  |  |
| Mean defecation frequency at baseline | 0.94 (0.79 – 1.12) | .459 | 0.93 (0.76 – 1.13) | .469 |
| Mean defecation consistency at baseline | 1.56 (0.46 – 5.34) | .475 |  |  |
| Mean nausea at baseline | 1.00 (0.86 – 1.16) | .994 |  |  |
| Mean headache at baseline | 0.99 (0.86 – 1.14) | .842 |  |  |
| Mean use of painkillers at baseline | 1.05 (0.87 – 1.28) | .608 |  |  |
| Mean school absence at baseline | 0.94 (0.76 – 1.16) | .573 |  |  |
| Mean loss of appetite at baseline | 0.94 (0.80 – 1.09) | .393 | 0.94 (0.78 – 1.12) | .477 |
| Child’s expectancy of treatment at baseline | 1.08 (0.91 – 1.28) | .406 | 1.00 (1.00 – 1.00) | .079 |
| Mother’s/parent 1 expectancy of treatment at baseline | 1.00 (0.81 – 1.24) | .980 |  |  |
| Father’s/parent 2 expectancy of treatment at baseline | 1.03 (0.86 – 1.22) | .780 |  |  |
| * For continuous predictors the OR should be interpreted as the change in OR with one unit increase in the predictor value. | | | | |

**Supplementary table 3** Logistic regression analysis to identify specific symptom improvement associated with treatment success at T1.

| Predictor* | Simple logistic regression | | Multiple logistic regression | |
| --- | --- | --- | --- | --- |
|  | **OR (95% CI)** | **p-value** | **OR (95% CI)** | **p-value** |
| Change in mean abdominal pain frequency, hours | 1.30 (1.10 – 1.54) | .003 | 1.27 (1.06 – 1.51) | .009 |
| Change in mean bloating severity | 1.35 (1.10 – 1.67) | .004 | 1.24 (0.94 – 1.64) | .119 |
| Change in mean flatulence severity | 1.33 (1.05 – 1.67) | .016 | 1.17 (0.89 – 1.56) | .261 |
| Change in mean defecation frequency | 1.00 (0.99 – 1.01) | .892 | 1.00 (0.99 – 1.01) | .910 |
| Change in mean defecation consistency | 2.31 (0.63 – 8.54) | .21 | 1.53 (0.36 – 6.63) | .563 |
| Change in mean headache severity | 1.20 (1.02 – 1.42) | .029 | 1.15 (0.94 – 1.41) | .180 |
| Change in mean nausea severity | 1.26 (1.04 – 1.53) | .017 | 1.10 (0.86 – 1.40) | .441 |
| Change in mean loss of appetite severity | 1.13 (0.94 – 1.36) | .196 | 1.02 (0.81 – 1.30) | .852 |
| * For continuous predictors the OR should be interpreted as the change in OR with one unit increase in the predictor value. | | | | |

**Supplementary table 4** Logistic regression analysis to identify baseline factors predicting adequate relief at T1.

| Predictor* | Simple logistic regression | | Multiple logistic regression | |
| --- | --- | --- | --- | --- |
|  | **OR (95% CI)** | **p-value** | **OR (95% CI)** | **p-value** |
| Sex (female vs male) | 2.50 (1.13– 5.54) | .025 | 1.18 (1.06 – 1.31) | .003 |
| Age at inclusion (in years) | 1.12 (0.93 – 1.34) | .221 |  |  |
| Ethnicity  White (reference)  Middle Eastern/North African  Afro-Caribbean  Other | 1.03 (0.89 – 1.19)  1.25 (1.04 – 1.50)  1.20 (1.00 – 1.44) | .708  .014  .052 | 0.97 (0.81 – 1.67)  1.10 (0.89 – 1.37)  1.01 (0.79 – 1.29) | .770  .375  .929 |
| First-line family member with FAPD | 0.87 (0.46 – 1.64) | .669 |  |  |
| Diagnosis (IBS vs. FAP-NOS) | 1.45 (0.75 – 2.82) | .272 |  |  |
| IBS subtype  IBS-U (ref)  IBS-C  IBS-D  IBS-M | 1.06 (0.89 – 1.29)  1.11 (0.92 – 1.34)  1.19 (0.97 – 1.45) | .511  .274  .094 | 1.09 (0.92 – 1.30)  1.11 (0.93 – 1.33)  1.17 (0.97 – 1.41) | .301  .239  .109 |
| Duration of symptoms (in years) | 1.03 (0.96 – 1.10) | .416 |  |  |
| Including center (academic vs teaching center) | 1.47 (0.80 – 2.70) | .212 |  |  |
| Mean abdominal pain severity at baseline | 0.44 (0.31 – 0.63) | <.001 | 0.90 (0.85 – 0.95) | <.001 |
| Mean abdominal pain frequency at baseline | 0.85 (0.75 – 0.95) | .006 | 1.00 (0.99 – 1.00) | .212 |
| Mean bloating at baseline | 0.95 (0.83 – 1.08) | .441 |  |  |
| Mean flatulence at baseline | 1.01 (0.88 – 1.17) | .919 |  |  |
| Mean defecation frequency at baseline | 1.11 (0.90 – 1.36) | .326 |  |  |
| Mean defecation consistency at baseline | 0.30 (0.06 – 1.44) | .131 |  |  |
| Mean nausea at baseline | 0.95 (0.80 – 1.13) | .577 |  |  |
| Mean headache at baseline | 0.94 (0.80 – 1.10) | .417 |  |  |
| Mean use of painkillers at baseline | 1.15 (0.93 – 1.42) | .188 |  |  |
| Mean school absence at baseline | 0.96 (0.74 – 1.25) | .767 |  |  |
| Mean loss of appetite at baseline | 1.03 (0.88 – 1.21) | .684 |  |  |
| Child’s expectancy of treatment at baseline | 1.23 (1.00 – 1.53) | .052 |  |  |
| Mother’s/parent 1 expectancy of treatment at baseline | 1.17 (0.93 – 1.47) | .194 |  |  |
| Father’s/parent 2  expectancy of treatment at baseline | 1.01 (0.84 – 1.21) | .923 |  |  |
| * For continuous predictors the OR should be interpreted as the change in OR with one unit increase in the predictor value. | | | | |

**Supplementary table 5** All adverse events

| Adverse events | N = 325 |
| --- | --- |
| No. of patients (%) | |
| Adverse events | |
| All adverse events | 13 (4%) |
| Serious adverse events | 0 (0%) |
| All adverse events ⱡ | |
| Gastroenteritis | 5 (1.5%) |
| Urinary tract infection | 2 (0.6%) |
| Fatigue | 2 (0.6%) |
| Dermatitis | 1 (0.3%) |
| Viral infection - unspecified | 1 (0.3%) |
| Headache | 1 (0.3%) |
| COVID-19 infection | 1 (0.3%) |

*ⱡ Adverse events are listed in descending order of frequency.*
