# Supplementary material for: The low FODMAP diet in adolescents functional abdominal in a non-guided setting: a prospective multicenter cohort study
Source: Eur J Pediatr. 2025 Feb 11;184(2):189. doi: 10.1007/s00431-025-05999-9 (PMC11814023; doi:10.1007/s00431-025-05999-9)
Supplement: Supplementary file 2 — Supplementary file2 (PDF 3.14 KB) [file 431_2025_5999_MOESM2_ESM.pdf]

Patiënteninformatie

Richtlijnen FODMaP-beperkt dieet

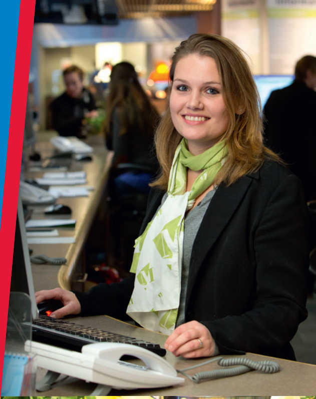

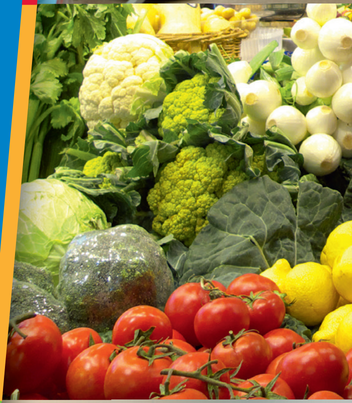

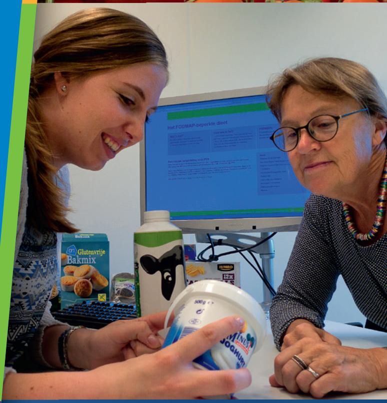

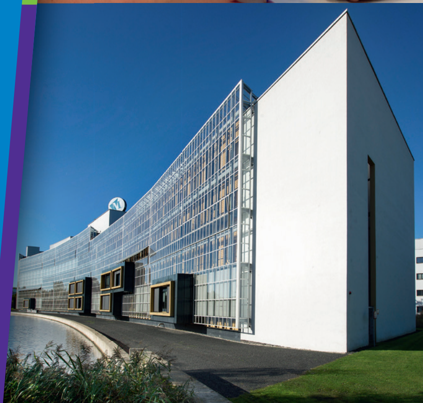

Richtlijnen FODMaP-beperkt dieet

Inleiding

Het FODMaP-beperkte dieet is ontwikkeld in Australië voor patiënten met ernstige klachten als gevolg van het prikkelbare darm syndroom (PDS), waarbij andere therapieën onvoldoende hebben geholpen. Een opgeblazen gevoel, buikpijn en een verstoorde stoelgang met diarree en/of obstipatie zijn symptomen die vaak voorkomen bij PDS. De precieze oorzaak van PDS is niet bekend. Veranderingen in leefstijl en dieet zoals een regelmatig en evenwichtig eetpatroon kunnen helpen in de behandeling bij PDS.

Deze brochure is opgesteld door de afdeling Diëtetiek en is bedoeld als aanvulling op de mondelinge informatie die u ontvangt van de diëtist.

Wat zijn FODMaPs?

FODMaPs zijn kleine moleculen die slecht of niet opgenomen worden in de dunne darm en in de dikke darm terechtkomen. In de dikke darm bevinden zich veel bacteriën.

De FODMaPs worden snel en in zeer grote hoeveelheden door de bacteriën gefermenteerd (=opgegeten). Hierbij komt gas vrij waardoor symptomen zoals een opgeblazen gevoel en winderigheid ontstaan. Symptomen zoals diarree of een verstoorde stoelgang ontstaan doordat er meer vocht wordt aangetrokken in de dunne en dikke darm.

Door producten met veel FODMaPs in de voeding te beperken komen er minder van deze moleculen in de dikke darm. Niet iedereen is even gevoelig. In de praktijk geven geringe hoeveelheden FODMaPs geen klachten.

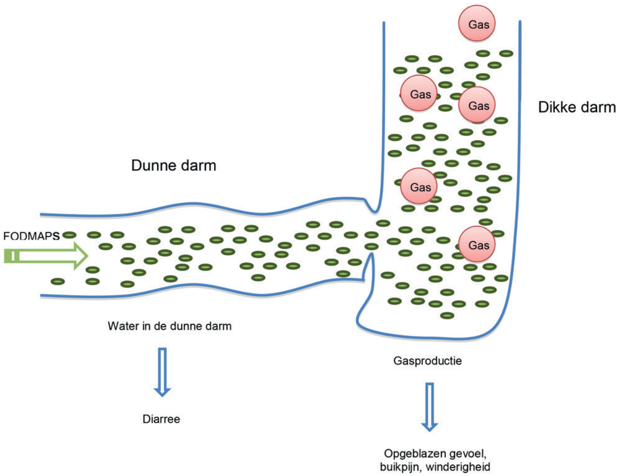

Niet geresorbeerde FODMAPs trekken extra water aan in de dunne en dikke darm. Er ontstaan gassen doordat bacteriën in de dikke darm de FODMAPs opeten (fermenteren).

F Fermenteerbare.
O Oligosachariden (fructanen en galacto-oligosachariden). Deze komen voor in peulvruchten en tarwe.
D Disachariden (lactose). Deze komen voor in melkproducten.
M Monosachariden (fructose). Deze komen voor in fruit en groenten.
a and.
P Polyolen (suiker alcoholen). Deze komen voor in zoetstoffen, fruit en groenten.

Dieet in de praktijk

FODMaPs zitten in heel veel levensmiddelen die dagelijks worden gegeten.
Het doel van het dieet is om de totale hoeveelheid van deze kleine moleculen (FODMaPs) die in de dikke darm komen te verminderen. Een kleine hoeveelheid FODMaPS in de voeding is niet erg omdat dit in de praktijk geen klachten veroorzaakt. Daarom wordt alleen het eten van FODMaP-rijke voedingsmiddelen afgeraden. Het dieet wordt dan ook 'het FODMaP-beperkte dieet' genoemd.

Hoe lang is het dieet nodig?

Om de klachten te verminderen is het nodig om het FODMaP-beperkte dieet 6 weken nauwkeurig te volgen. De mate van gevoeligheid voor FODMaPs, met

name voor mono- en disachariden en polyolen, kan erg verschillen. Het is goed mogelijk dat bij u niet alle FODMaPs klachten geven. Na de 6-weekse proefperiode van het dieet zal er, indien het dieet bij u voldoende effect lijkt te hebben, samen met de diëtist onderzocht worden welke voedingsmiddelen u wel kunt verdragen.

Is het dieet gezond?

Ondanks de beperkingen in het FODMaP-beperkte dieet is het goed mogelijk een gezonde voeding te kiezen. De folder van het Voedingscentrum met de *Schijf van Vijf* kan hierbij een hulpmiddel zijn.

Etiketten lezen

Op het etiket is veel informatie over een product terug te vinden. Fabrikanten zijn namelijk verplicht om ingrediënten te melden op hun producten. Alle ingrediënten worden in afnemende volgorde van gewicht vermeld met uitzondering van mengsels van groenten en fruit. Dus hoe eerder een ingrediënt wordt genoemd, hoe meer er van dat ingrediënt in het product zit. Een product kan van samenstelling veranderen in verloop van tijd, blijf daarom regelmatig de etiketten controleren.

Ingrediënten die vermeden dienen te worden zijn niet altijd makkelijk te herkennen op het etiket. Onderstaande lijst kan daarbij helpen.

FODMaPs	Ingrediënten
Fructaan	Inuline
FOS	Oligofructose
Fructose	Fructose, fructose siroop, glucose-fructose siroop en fructose mais siroop
Lactose	Lactose, botermelk, melkbestanddelen, melkpoeder en wei
Polyolen	Isomalt (E953), lactitol (E966), maltitol (E965), mannitol (E421), sorbitol (E420) en xylitol (E967)

Daarnaast is de fabrikant verplicht om de bekende allergene stoffen specifiek te noemen. Van de FODMaPs zijn dit alleen gluten- en lactose bevattende ingrediënten.

Lijst met FODMaP-arme en FODMaP-rijke producten

	FODMaP-arm	FODMaP-rijk
**Graanproducten**		
Brood en Brood-vervangers	• beschuit (2 stuks)<br>• brood glutenvrij** en tarwezetmeelvrij<br>• brood gemaakt van 100% spelt*<br>• zuurdesem speltbrood gemaakt van 100% spelt*<br>• crackers glutenvrij**<br>• croissants glutenvrij**<br>• havermout<br>• knäckebröd glutenvrij**<br>• maiswafel<br>• maisvlokken/Cornflakes<br>• quinoavlokken<br>• rijstwafel<br>• tarwebiscuit (2 stuks)	• brood gemaakt van tarwe, rogge, meergranen en gerst<br>• meer dan 2 snee speltbrood per maaltijd<br>• beschuit<br>• ciabatta<br>• croissants<br>• focaccia<br>• knäckebröd<br>• muesli gemaakt van tarwe, meergranen en fruit<br>• naan brood<br>• roggebrood<br>• rijstwafel met meergranen<br>• tarwevlokken
Bloem/meel gemaakt van	• aardappel<br>• boekweit<br>• cassave (tapioca)<br>• gierst<br>• haver<br>• mais (ook maizena en custardpoeder)<br>• polenta<br>• quinoa<br>• rijst<br>• teff	• amandel<br>• amaranth<br>• gerst<br>• khorasantarwe (kamut)<br>• rogge<br>• spelt<br>• tarwe<br>• tarwerogge<br>• tarwegriesmeel
Rijst	• alle soorten rijst b.v. Basmati, Pandan, Risotto rijst<br>• witte rijst<br>• zilvervlies rijst	• bulgur<br>• couscous<br>• meergranen rijst
Pasta droog en vers	• boekweitnoedels (soba) van 100% boekweit<br>• kelpnoedels<br>• pasta glutenvrij<br>• mihoen<br>• noedels van rijst<br>• quinoapasta	• pasta, glutenvrij met kikker-erwtenmeel<br>• pasta van spelt<br>• pasta van tarwe<br>• gnocchi van tarwe
Overige deegwaren	• pizzabodem glutenvrij<br>• taco van maismeel<br>• tortilla van maismeel<br>• wrap van maismeel	• pizza's<br>• producten van bladerdeeg<br>• taco, tortilla, wrap van tarwebloem

* Kies voor brood gemaakt van puur speltmeel. Een mix van speltmeel met andere granen, zoals tarwebloem is niet geschikt. Om een goed brood te bakken van spelt wordt vaak een kleine hoeveelheid tarwegluten toegevoegd. Dit is geen probleem.

** Merken van glutenvrije producten zijn bijvoorbeeld Consenza, Proceli en Schär.

	FODMaP-arm	FODMaP-rijk
**Groente**		
	• agar agar<br>• alfalfa<br>• andijvie<br>• aubergine<br>• augurk<br>• bamboe scheuten<br>• champignons uit blik<br>• chili peper<br>• courgette<br>• gember<br>• kappertjes<br>• komkommer<br>• koolraap<br>• knolselderij<br>• lente ui, groene deel<br>• maiskolf<br>• meiknol<br>• oesterzwammen<br>• okra (beperkt)<br>• olijven<br>• paksoi<br>• paprika, rode<br>• paprika, groene (beperkt)<br>• pastinaak<br>• pompoen, oranje Hokkaido<br>• pompoen, spaghetti<br>• prei, groene deel<br>• rabarber<br>• radijs<br>• rucola<br>• sellerie knol<br>• sla<br>• snijbiet<br>• spinazie<br>• taugé<br>• tomaat<br>• witlof<br>• wortel<br>• zeewier (nori)	• artisjok<br>• asperge<br>• avocado<br>• bleekselderij<br>• bloemkool<br>• champignons<br>• doperwten<br>• knoflook<br>• kool – alle soorten behalve rode en witte kool<br>• lente ui, witte deel<br>• mais uit blik<br>• paddenstoelen<br>• peultjes<br>• pompoen, fles<br>• prei, witte deel<br>• rode biet<br>• sjalot<br>• spruitjes<br>• sugar snaps<br>• tuinbonen<br>• ui<br>• zongedroogde tomaten<br>• zuurkool
Groente maximaal 1 opscheplepel	• broccoli<br>• kool, witte<br>• kool, rode<br>• paprika, groene<br>• sperziebonen<br>• venkel	

	FODMaP-arm	FODMaP-rijk
**Aardappelen**		
	• aardappelen<br>• aardappel (gnocchi)	• zoete aardappelen

	FODMaP-arm	FODMaP-rijk
**Fruit**		
	• aardbei<br>• ananas<br>• banaan(⅓)<br>• bosbes<br>• carambola (sterfruit)<br>• citroen<br>• doerian<br>• druiven<br>• framboos<br>• grapefruit (¼)<br>• guave<br>• kiwi (green & gold)<br>• kokosnoot<br>• kumkwats<br>• limoen<br>• mandarijn<br>• meloen Galia, honing en Cantaloupe<br>• mineola<br>• papaya<br>• passievrucht<br>• pitahaya (drakenfruit)<br>• rabarber<br>• sinaasappel	Onderstaande fruitsoorten zowel vers, in blik als gedroogd<br>• aalbes<br>• abrikoos<br>• appel<br>• braam<br>• granaatappel<br>• kers<br>• lychee<br>• mango<br>• nectarine<br>• peer, alle soorten<br>• perzik, alle soorten<br>• pruim<br>• sharonfruit (kaki)<br>• sultana<br>• vijg<br>• watermeloen<br>Let bij producten met fruit op de vermelding 'fructose' op het etiket
**Fruit-gedroogd**		
	• banaan maximaal 1 handje vol<br>• cranberries –max. 1 eetlepel<br>• krenten-max. 1 eetlepel<br>• kokosnoot-max. 2 eetlepels<br>• rozijn – max. 1 eetlepel (geen sultana)	• abrikoos<br>• ananas<br>• appel<br>• dadel<br>• goji bes<br>• mango<br>• peer<br>• pruim<br>• sultana

Advies: neem per keer een kleine portie fruit

	FODMaP-arm	FODMaP-rijk
**Zuivel**		
Melk	• amandelmelk<br>• kokosmelk (beperkt)<br>• lactosevrije melk*<br>• rijstmelk<br>• chocolademelk van cacao en lactosevrije melk*	• alle soorten melk van dierlijke afkomst<br>• lactosevrije melk met FODMaP-rijke toevoegingen<br>• sojamelk op basis van sojabonen<br>• chocolademelk<br>• havermelk<br>• karnemelk<br>• kefir<br>• koffiemelk
Melkproducten	• kokosyoghurt<br>• lactosevrije producten zoals:<br>- lactosevrije kwark*<br>- lactosevrije pudding*<br>- lactosevrije yoghurt*<br>• sorbetijs<br>• waterijs	• kwark<br>• lactosevrije en sojaprodukten met FODMaP-rijke toevoegin-gen<br>• yoghurt, desserts op basis van sojabonen<br>• room zoals:<br>- crème fraîche<br>- slagroom<br>- zure room (sour cream)<br>• vla<br>• yoghurt<br>• ijs (slagroomijs, yoghurtijs)<br>• probiotica**
Kaas	• alle harde kazen, zoals Cheddar, Goudse, Edammer, Emmenthaler<br>• gerijpte kazen zoals Brie Camembert, Feta, Mozzarella (beperkt)	alle zachte kazen zoals:<br>• geitenkaas<br>• hüttenkäse/ cottage cheese<br>• ricotta<br>• roomkaas<br>• schapenkaas<br>• smeerkaas<br>• smeltkaas<br>• verse kaas

* Lactosevrije melk bijvoorbeeld van het merk Campina, huismerk AH en MinusL (dit is koemelk waar lactose uit verwijderd is).

** Probiotica producten bijvoorbeeld van het merk Yakult en Actimel.

	FODMaP-arm	FODMaP-rijk
**Eieren, gevogelte, vlees, vis, vleesvervangers**		
	• eieren<br>• gevogelte onbewerkt<br>• vlees onbewerkt<br>• vleeswaren<br>• vis en schaaldieren onbewerkt<br>• vegetarische producten op basis van:<br>- quorn<br>- sojaeiwit<br>- tahoe/ tofu en tempeh	• bewerkte producten waar veel tarwe in is verwerkt zoals:<br>- gehaktbal en schnitzel<br>- kipnuggets, en kipschnitzel<br>- vissticks, visburger<br>• vegetarische producten die tarwe, lactose , ui of knoflook bevatten*

* Vegetarische producten die tarwe en lactose bevatten bijvoorbeeld van het merk Valess of Tivall.

	FODMaP-arm	FODMaP-rijk
**Peulvruchten**		
	• linzen, uit blik of pot, maximaal 3 eetlepels<br>• mungbonen (beperkt)<br>• tempeh<br>• tofu	• bruine bonen<br>• flageolets<br>• kapucijners<br>• kidneybonen<br>• kikkererwten<br>• limabonen<br>• linzen<br>• sojabonen<br>• spliterwten<br>• witte bonen

	FODMaP-arm	FODMaP-rijk
**Zoetmiddelen**		
	• (kristal)suiker<br>• poedersuiker<br>• palmsuiker<br>• rietsuiker<br>• ahornsiroop (maple)<br>• maltosesiroop<br>• zoetstoffen: op basis van stevia, aspartaam, saccharine<br>• dextrose<br>• glucose	• isomalt<br>• lactitol<br>• maltitol<br>• mannitol<br>• sorbitol<br>• xylitol<br>• agavesiroop<br>• honing<br>• fructose

	FODMaP-arm	FODMaP-rijk
**Kruiden en specerijen**		
	• alle soorten verse en gedroogde kruiden en specerijen<br>• asafoetidapoeder	• knoflookpoeder<br>• kruidenmixen met ui, knoflook<br>• uipoeder

	FODMaP-arm	FODMaP-rijk
**Noten en zaden**		
	Per keer maximaal een klein handje van:<br>• kastanje<br>• kokosnoot<br>• macadamia<br>• paranoten<br>• pecannoten<br>• pinda's<br>• pitten van pompoen, pijnboom en zonnebloem<br>• walnoten<br>• zaden: chiazaad, lijnzaad, maanzaad en sesamzaad<br>• amandelen < 10<br>• hazelnoten < 10	• pistachenoten<br>• cashewnoten

	FODMaP-arm	FODMaP-rijk
**Overige**		
Dranken	• frisdranken zonder sorbitol en xylitol<br>• koffie en thee zonder melk<br>• sappen van FODMaP-arm fruit (puur sap 100 ml per keer) let op dat ze niet aangelengd zijn met appelsap<br>• ranja*<br>• ijsthee**<br>• tomatensap<br>• wortelsap	• koffie van cichorei<br>• thee van chai, kamille, paardenbloem of venkel<br>• kruidenthee FODMaP-rijk<br>• sappen FODMaP-rijk fruit zoals appel, peer, mango, tropische vruchten<br>• sappen met groenten zoals ui en bieten<br>• frisdranken gezoet met xylitol en/of sorbitol
Dranken alcohol-houdend	Alcohol beperkt:<br>• bier<br>• gin<br>• vodka<br>• whiskey<br>• wijn rood/ wit	• cider<br>• dessertwijn of zoete wijn<br>• port<br>• rum<br>• rijstwijn<br>• likeuren
Broodbeleg	• chocolade (puur) producten<br>• jam van FODMaP-arm fruit<br>• pindakaas<br>• kaas: zie tabel melkproducten<br>• stroop van riet- of bietsuiker<br>• vleeswaren, zie tabel vlees	• appel en perenstroop<br>• honing<br>• jam gezoet met sorbitol, xylitol, fructose en van FODMaP-rijk fruit
Koek, gebak en snoep	• glutenvrij koek en gebak of gemaakt van spelt en zonder FODMaP-rijke toevoegingen<br>• snoep zoals bijvoorbeeld chocola puur, drop en pepermunt	• koek en gebak<br>• suikervrije producten zoals bijvoorbeeld kauwgom en mints met sorbitol en xylitol
Snacks	• chips op basis van aardappel, cassave of mais<br>• kroepoek<br>• patat frites<br>• popcorn	• snacks waar veel tarwe in is verwerkt zoals bijvoorbeeld:<br>- bitterbal, kroket, frikandel, hamburger<br>- lekkerbekje<br>- nasischijf<br>- oliebol<br>• producten met ui of knoflook

* Ranja van bijvoorbeeld Slimpie sinaasappel, let op dat ranja geen appelsap en/of fructose siroop bevat.

** IJsthee van bijvoorbeeld PickWick, Iced tea with lemon, let op dat het product geen fructose (-corn) siroop bevat.

	FODMaP-arm	FODMaP-rijk
**Overige**		
Sauzen en smaakmakers	• azijn<br>• chilisaus<br>• ketjap, oestersaus, sojasaus<br>• mayonaise<br>• mosterd<br>• tobascosaus<br>• tomatenketchup of puree<br>• wasabi<br>• worcestershiresaus	• meeste dressings, slasauzen<br>• alle sauzen, smaakmakers waarin ui, knoflook, honing, fructose, tarwebloem of melkbestanddelen is verwerkt
Vetten	• bak en braadproducten<br>• halvarine en margarine<br>• olie, alle soorten	• roomboter (in grote hoeveel-heden)

Tips voor de maaltijden

Ontbijt:

- Pap van havermout met lactosevrije melk.
- Cornflakes met lactosevrije melk of yoghurt.
- Sneetjes speltbrood met halvarine en belegd met aardbeienjam en kipfilet.
- Fruit zoals een kiwi of sinaasappel.

Broodmaaltijd:

- Tosti van speltbrood of glutenvrij brood met halvarine, ham en kaas.
- Maak eens een uitsmijter met speltbrood of glutenvrij brood, ei, ham en tomaat. Breng op smaak met een snufje peper en zout.
- Pannenkoeken gemaakt van boekweitmeel met poedersuiker of stroop van riet- of bietsuiker.

Warme maaltijd:

- Bouillon of (zelfgemaakte) soep gemaakt van vlees en FODMaP-arme groenten zoals tomaat, stukjes wortel, paprika, sperzieboontjes, groen van prei en gebonden met rijst.
- Aardappels, rijst, mihoen of glutenvrije pasta.
- Serveer met groenten zoals sperziebonen, sla, spinazie, worteltjes of witlof.
- Een stukje vlees, vis, ei of kaas, bereid in olie of bak- en braadproduct.
- Schnitzel of lekkerbekje maken? Wentel vlees of vis eerst door een ei en wentel het vervolgens door fijngemaakte cornflakes, kruim van speltbrood of glutenvrije paneermeel.
- Jus gemaakt van vleesnat of bouillon, gebonden met maïzena.
- Saus voor bij rijst of de glutenvrije pasta, neem voor de saus als basis een blik tomatenpuree, passata of kokosnootmelk.
- Toetje gemaakt van lactosevrije melk, custardpoeder en suiker.
- Schaaltje fruit, bijvoorbeeld aardbeien.

Tussendoor:

- Fruit of groenten zoals druiven, sinaasappel, snoeptomaat of snackkomkommer.
- Een handje pinda's, klein schaaltje popcorn of chips.
- Een paar koekjes zoals avenas van Dr. Schär of vanille koffiekoekjes van 3Pauly.
- Rijstwafels belegd met kaas of chocoladepasta.

Tips voor uit eten:

Om onbezorgd van uw maaltijd te kunnen genieten is enige voorbereiding nodig.

- Bekijk vooraf de menukaart om te zien of u voldoende keus hebt. Veel restaurants publiceren hun menu op de website.
- Neem vooraf contact op met het restaurant, vraag naar de mogelijkheden. Is een glutenvrije maaltijd mogelijk?
- Vraag of de salade aangemaakt kan worden met alleen wat olie en azijn.
- Kies voor gerechten zonder saus en voor vlees zonder paneerlaagje.
- Producten als pizza en pasta zijn niet geschikt bij het dieet vanwege de tarwe. Toch naar de pizzeria? Kies dan voor een Italiaans restaurant dat ook andere gerechten maakt of vraag naar glutenvrije pizza/pasta en kies uw eigen vulling.
- Houdt u van Chinees eten? Een wok-restaurant is heel geschikt, omdat u daar zelf uw ingrediënten kunt kiezen.

Veel gestelde vragen

Kan ik een keertje zondigen?

Het kan voorkomen dat u per ongeluk een product met veel FODMaPs binnen krijgt, of er niet onderuit kan een keer te 'zondigen'. Hoewel dit bij u enkele dagen de buikklachten kan verergeren, zal er geen schade aan de darm ontstaan.

Wat als voedingsmiddelen niet in de lijst staan?

De lijst kan niet volledig zijn omdat van veel producten de samenstelling onvoldoende bekend is. Ook komen er voortdurend nieuwe producten op de markt. Heeft u vragen over een bepaald product, bespreek dit dan met uw diëtist.

Wat als mijn klachten niet verbeteren?

Het kan voorkomen dat het nog niet beter gaat, hoewel u het dieet nauwkeurig toepast. Het dieet werkt niet bij alle patiënten. Ga dan naar uw diëtist om te bespreken hoe het verder moet.

Waarom moet ik glutenvrij brood eten. Ik heb toch geen glutenintolerantie?

Tarwe, gerst en rogge kunnen bij PDS patiënten klachten geven. Deze granen bevatten veel gluten en glutenvrije producten bevatten deze graansoorten niet. Glutenvrije producten kunnen daarom binnen het FODMaP-beperkte dieet een

goede keuze zijn. Maar ook speltbrood dat wel gluten maar geen tarwe bevat past binnen het FODMaP-beperkte dieet. Let wel op dat er geen tarwezetmeel in is verwerkt.

Waar koop ik voedingsmiddelen vrij van gluten en lactose?

Glutenvrije en lactosevrije producten zijn onder andere te koop bij de grotere supermarkten, reformzaken en webwinkels.

Mijn arts heeft voedingsvezels aanbevolen vanwege obstipatieklachten. Kan ik die gebruiken?

Voedingsvezels, waaronder prebiotica zoals inuline, kunnen veel oligosachariden bevatten en dus klachten veroorzaken bij PDS. Psylliumvezels worden over het algemeen goed verdragen. Overleg met uw diëtist.

Ik heb gelezen dat Yakult goed is voor de darmen. Hoe zit dat?

Producten als Yakult en Actimel zijn zogenaamde probiotica. Dit zijn melkdranken waaraan bepaalde bacteriestammen zijn toegevoegd waarvan sommigen beweren dat die de darmflora gunstig beïnvloeden. Het wetenschappelijk bewijs hiervoor ontbreekt echter. Omdat deze dranken ook lactose bevatten zijn ze niet geschikt voor het FODMaP-beperkte dieet. Desgewenst kan na de proefperiode van 6 weken het gebruik van probiotica in overleg met uw diëtist geprobeerd worden.

Tot slot

Heeft u na het lezen van deze brochure nog vragen over het dieet, dan kunt u een email sturen naar: fodmap@mzh.nl of bezoek onze website www.mzh.nl/pds

Heeft u een afspraak en bent u verhinderd? Neem dan contact op met de afdeling Diëtetiek, tel: (050) 524 5136 (maandag t/m vrijdag tussen 8.30 en 9.00 uur).

Datum: ....

Naam diëtist: ....

Links naar websites voor meer informatie over PDS of voeding:

Maag Lever Darm Stichting	www.mlds.nl
Voedingscentrum	www.voedingscentrum.nl
PDS belangenvereniging	www.pdsb.nl

De app LOW FODMAP diet van the Monash University is beschikbaar via de Google PlayStore of de AppStore. Dit is een Engelstalige app waaraan eenmalig kosten zijn verbonden.

© 2019 Martini Ziekenhuis. Als u iets wilt doen met de inhoud van deze brochure, neem dan contact op met de afdeling Diëtetiek van het Martini Ziekenhuis.

Ruimte voor eigen aantekeningen

Martini Ziekenhuis

Postadres
Postbus 30033
9700 RM Groningen

Bezoekadres
Van Swietenplein 1
Groningen

Algemeen telefoonnummer
(050) 524 52 45

www.martiniziekenhuis.nl

03-2019
20140015

een santeon ziekenhuis
