# Supplementary material for: The low FODMAP diet in adolescents functional abdominal in a non-guided setting: a prospective multicenter cohort study
Source: Eur J Pediatr. 2025 Feb 11;184(2):189. doi: 10.1007/s00431-025-05999-9 (PMC11814023; doi:10.1007/s00431-025-05999-9)
Supplement: Supplementary file 3 — Supplementary file3 (PDF 212 KB) [file 431_2025_5999_MOESM3_ESM.pdf]

Lijst met FODMaP-arme en FODMaP-rijke producten

Graanproducten	FODMaP-arm	FODMaP-rijk
Brood en Broodvervangers	• Brood glutenvrij, teffvrij en tarwezetmeelvrij<br>• Brood gemaakt van speltmeel en tarwevrij*<br>• Crackers glutenvrij en teffvrij<br>• Croissants glutenvrij en teffvrij<br>• Havermout<br>• Knäckebröd glutenvrij en teffvrij<br>• Maiswafel<br>• Maisvlokken /Cornflakes<br>• Rijstwafel	• Brood gemaakt van tarwe, rogge, meergranen en gerst<br>• Beschuit<br>• Ciabatta<br>• Croissants<br>• Focaccia<br>• Knäckebröd<br>• Muesli gemaakt van tarwe, meergranen en fruit<br>• Naan brood<br>• Roggebrood<br>• Rijstwafel met meergranen<br>• Tarwevlokken
Bloem/meel gemaakt van:	• Aardappel<br>• Boekweit<br>• Haver<br>• Mais<br>• Polenta<br>• Quinoa<br>• Rijst<br>• Spelt	• Gerst<br>• Rogge<br>• Tarwe<br>• Tarwerogge<br>• Tarwegriesmeel
Rijst	• Alle soorten rijst b.v. Basmati, Pandan, Risotto rijst<br>• Witte rijst<br>• Zilvervlies rijst	• Bulgur<br>• Couscous<br>• Meergranen rijst
Pasta droog en vers	• Pasta glutenvrij<br>• Pasta van puur spelt<br>• Mihoen	• Pasta van tarwe<br>• Gnocchi
Overige deegwaren	• Pizza glutenvrij<br>• Tortilla van maismeel	• Pizza’s<br>• Producten van bladerdeeg<br>• Tortilla, wrap van tarwebloem
Aardappelen	• Aardappelen	• Zoete aardappelen
		• Bruine bonen

Peulvruchten		• Flageolets<br>• Kapucijners<br>• Kidneybonen<br>• Kikkererwten<br>• Limabonen<br>• Linzen<br>• Sojabonen<br>• Spliterwten<br>• Witte bonen

* Kies voor brood gemaakt van puur speltmeel. Een mix van speltmeel met andere granen is niet geschikt. Om een goed brood te bakken van spelt wordt vaak een kleine hoeveelheid tarwegluten toegevoegd. Dit is geen probleem. Tarwebloem is niet toegestaan.
**Merken van glutenvrije producten zijn bijvoorbeeld Consenza, Proceli en Schär.

Groente	FODMaP-arm	FODMaP-rijk
	• Alfalfa<br>• Andijvie<br>• Aubergine<br>• Bamboe scheuten<br>• Broccoli (beperkt)<br>• Chili peper<br>• Courgette<br>• Gember<br>• Komkommer<br>• Lente ui, groene deel<br>• Mais (beperkt)<br>• Olijven<br>• Paksoi<br>• Pastinaak<br>• Paprika<br>• Pompoen<br>• Prei, groene deel<br>• Rabarber<br>• Radijs<br>• Sellerie knol<br>• Sla<br>• Sperziebonen<br>• Spinazie<br>• Taugé<br>• Tomaat<br>• Venkel (beperkt)<br>• Witlof<br>• Wortel	• Artisjok<br>• Asperge<br>• Avocado<br>• Bleekselderij<br>• Bloemkool<br>• Champignons<br>• Doperwten<br>• Knoflook<br>• Kool – alle soorten-<br>• Lente ui, witte deel<br>• Okra<br>• Peultjes<br>• Prei, witte deel<br>• Rode biet<br>• Sjalot<br>• Spruitjes<br>• Sugar snaps<br>• Ui
Fruit	• Aardbei<br>• Ananas	Onderstaande fruitsoorten zowel vers, in blik als gedroogd

	• Banaan<br>• Bosbes<br>• Citroen<br>• Druiven (niet meer dan 10)<br>• Framboos<br>• Grapefruit (¼)<br>• Kiwi<br>• Limoen<br>• Mandarijn<br>• Meloen Galia en Cantaloupe<br>• Papaya<br>• Passievrucht<br>• Sinaasappel	• Abrikoos<br>• Appel<br>• Braam<br>• Kers<br>• Lychee<br>• Mango<br>• Nectarine<br>• Peer<br>• Perzik<br>• Pruim<br>• Watermeloen<br>Let bij producten met fruit op de etiketten de vermelding “fructose”

Advies: neem per keer een kleine portie fruit

Kruiden	• alle overige soorten	• Knoflookpoeder<br>• Poeder met ui<br>• Kaneel, grote hoeveelheden
Noten	• Alle andere soorten, maximaal een klein handje<br>• Amandelen < 10<br>• Hazelnoten < 10	• Pistachenoten<br>• Cashewnoten

Zuivel	FODMaP-arm	FODMaP-rijk
Melk	• Lactosevrije melk*<br>• Rijstmelk<br>• Sojamelk**	• Alle soorten melk van dierlijke afkomst<br>• Lactosevrije melk en sojamelk met FODMaPrijke toevoegingen<br>• Chocolademelk<br>• Karnemelk<br>• Kefir<br>• Koffiemelk
Melkproducten	• Lactosevrije producten zoals:<br>- lactosevrije kwark*<br>- lactosevrije pudding*<br>- lactosevrije yoghurt*	• Kwark<br>• Lactosevrije en sojaprodukten met FODMaPrijke toevoegingen<br>• Room zoals:

	• Room op basis van soja**<br><br><br>• Vla op basis van soja**<br>• Yoghurt op basis van soja**<br>• Waterijs	- crème fraîche<br>- slagroom<br>- zure room (sour cream)<br>• Vla<br>• Yoghurt<br>• IJs (slagroomijs, yoghurtijs)<br>• Probiotica***
Kaas	• Alle harde kazen, zoals Cheddar, Goudse, Edammer, Emmenthaler<br>• Gerijpte kazen zoals Brie Camembert, Feta, Mozzarella (beperkt)	• Alle zachte kazen<br>• Geitenkaas<br>• Hüttenkäse/ cottage cheese<br>• Ricotta<br>• Roomkaas<br>• Schapenkaas<br>• Smeerkaas<br>• Smeltkaas<br>• Verse kaas

* Lactosevrije melk bijvoorbeeld van het merk Campina, huismerk AH en MinusL (dit is koemelk waar lactose uit verwijderd is).
** Soja producten bijvoorbeeld van het merk Alpro.
*** Probiotica producten bijvoorbeeld van het merk Yakult en Actimel.

Eieren, gevogelte, vlees, vis, vleesvervangers	FODMaP-beperkt	FODMaP-rijk
	• Eieren<br>• Gevogelte onbewerkt<br><br>• Vlees onbewerkt<br>• Vleeswaren<br>• Vis en schaaldieren onbewerkt<br>• Vegetarische producten zoals quorn, tahoe, tofu en tempee	• Bewerkte producten waar tarwe in is verwerkt zoals kipnuggets, en kipschnitzel<br>• Gehakt bal en schnitzel<br><br>• Vissticks, visburger<br>• Vegetarische producten die tarwe en lactose bevatten*

* Vegetarische producten die tarwe en lactose bevatten bijvoorbeeld van het merk Valess of Tivall.

Zoetmiddelen	FODMaP-beperkt	FODMaP-rijk
	• Kristalsuiker<br>• Poedersuiker<br>• Zoetstoffen:op stevia, aspartaam, saccharine	• Isomalt<br>• Lactitol<br>• Maltitol<br>• Mannitol<br>• Sorbitol<br>• Xylitol

Overige	FODMaP-arm	FODMaP-rijk
Dranken	• Koffie en thee<br>• Sappen van FODMaP-arm fruit (puur sap 100 ml per keer)<br>Let op dat ze niet aangelengd<br>zijn met appelsap<br>• Frisdranken zonder sorbitol en xylitol<br>• Ranja*<br>• IJsthee**	• Koffie van cichorei<br>• Thee van paardebloem<br>• Kruidenthee FODMaP-rijk<br>• Sappen FODMaP-rijk fruit zoals appel, peer, mango, tropische vruchten<br>• Sappen met groenten zoals ui en bieten<br>• Frisdranken gezoet met xylitol en/of sorbitol
Dranken alcoholhoudend	Alcohol beperkt:<br>• Bier<br>• Gin<br>• Vodka<br>• Whiskey<br>• Wijn rood/ wit	• Cider<br>• Dessertwijn of zoete wijn<br>• Port<br>• Rum<br>• Rijstwijn<br>• Likeuren
Brood beleg	• Chocolade (puur) producten<br>• Jam van FODMaP-arm fruit<br>• Pindakaas<br>• Kaas: zie tabel melkproducten<br>• Stroop van riet- of bietsuiker<br>• Vleeswaren, zie tabel vlees	• Appel en perenstroop<br>• Honing<br>• Jam gezoet met sorbitol, xylitol en<br>van FODMaP-rijk fruit
Koek, gebak en snoep	• Glutenvrij koek en gebak of gemaakt van spelt en zonder FODMaPrijke toevoegingen<br>• Snoep zoals bijvoorbeeld	• Koek en gebak<br>• Suikervrije producten zoals bijvoorbeeld kauwgom en mints<br>met sorbitol en xylitol

	chocola puur, drop en pepermunt	
Snacks en Sauzen	• Chips op basis van aardappel, cassave of mais<br>• Kroepoek<br>• Popcorn<br><br>• Sauzen waarin geen tarwebloem is verwerkt	• Snacks waar tarwe in is verwerkt zoals bijvoorbeeld:<br>- bitterbal, kroket, frikandel, hamburger<br>- nasischijf<br>- oliebol<br>• producten met ui of knoflook<br>• Sauzen waarin tarwebloem of fructose siroop is verwerkt
Vetten	• Bak en braadproducten<br>• Halvarine en margarine<br>• Olie, alle soorten	• Roomboter (kleine hoeveelheid is geen probleem)

* Ranja van bijvoorbeeld Slimpie sinaasappel, let op dat ranja geen appelsap en/of fructose siroop bevat.
** Ijsthee van bijvoorbeeld Lipton, Iced tea with lemon, let op dat het product geen fructose (-corn) siroop bevat.
